# Alkaloid content variability in the seeds of narrow-leafed lupine accessions from the VIR collection under the conditions of the Russian Northwest

**DOI:** 10.18699/VJGB-23-17

**Published:** 2023-04

**Authors:** M.A. Vishnyakova, A.V. Salikova, T.V. Shelenga, G.P. Egorova, L.Yu. Novikova

**Affiliations:** Federal Research Center the N.I. Vavilov All-Russian Institute of Plant Genetic Resources (VIR), St. Petersburg, Russia; Federal Research Center the N.I. Vavilov All-Russian Institute of Plant Genetic Resources (VIR), St. Petersburg, Russia; Federal Research Center the N.I. Vavilov All-Russian Institute of Plant Genetic Resources (VIR), St. Petersburg, Russia; Federal Research Center the N.I. Vavilov All-Russian Institute of Plant Genetic Resources (VIR), St. Petersburg, Russia; Federal Research Center the N.I. Vavilov All-Russian Institute of Plant Genetic Resources (VIR), St. Petersburg, Russia

**Keywords:** narrow-leafed lupine, alkaloids, domestication traits, spontaneously dehiscent pods, nondehiscent pods, seed color, люпин узколистный, алкалоиды, признаки доместикации, спонтанно вскрывающийся боб, невскрывающийся боб, окраска семени

## Abstract

Alkaloid content was assessed in the seeds of 59 narrow-leafed lupine (Lupinus angustifolius L.) accessions from the VIR collection in the environments of Leningrad Province. The selected set included accessions of different statuses (wild forms, landraces, and advanced cultivars) and different years of introduction to the collection. Alkaloids were analyzed using gas-liquid chromatography coupled with mass spectrometry. Concentrations of main alkaloids: lupanine, 13-hydroxylupanine, sparteine, angustifoline and isolupanine, and their total content were measured. The total alkaloid content variability identified in the seeds of the studied set of accessions was 0.0015 to 2.017 %. In most cases, the value of the character corresponded to the accession’s status: modern improved cultivars, with the exception of green manure ones, entered the group with the range of 0.0015–0.052 %, while landraces and wild forms showed values from 0.057 to 2.17 %. It is meaningful that the second group mainly included accessions that came to the collection before the 1950s, i. e., before the times when low-alkaloid cultivars were intensively developed. Strong variability of the character across the years was observed in the accessions grown under the same soil and climate conditions in both years. In 2019, the average content of alkaloids in the sampled set was 1.9 times higher than in 2020. An analysis of weather conditions suggested that the decrease in alkaloid content occurred due to a significant increase in total rainfall in 2020. Searching for links between the content of alkaloids and the type of pod (spontaneously non-dehiscent, or cultivated, spontaneously dehiscent, or wild, and intermediate) showed a tendency towards higher (approximately twofold in both years of research) total alkaloid content in the accessions with the wild pod type and the nearest intermediate one compared to those with the pod non-dehiscent without threshing. The correlation between the average total alkaloid content and seed color, reduced to three categories (dark, or wild, light, or cultivated, and intermediate), was significantly stronger in the group with dark seeds (5.2 times in 2019, and 3.7 times in 2020). There were no significant differences in the percentage of individual alkaloids within the total amount either between the years of research or among the groups with different pod types or the groups with different seed coat colors.

## Abstract (ru)

Изучали содержание алкалоидов в семенах люпина узколистного (Lupinus angustifolius L.) у 59 образцов из коллекции ВИР в условиях Ленинградской области. В выборку были включены образцы разного статуса (дикие формы, староместные сорта, сорта научной селекции) и различных лет поступления в коллекцию. Алкалоиды определяли методом газожидкостной хроматографии, сопряженной с масс-спектрометрией. Определены концентрации основных алкалоидов в семенах: люпанина, 13-гидроксилюпанина, спартеина, ангустифолина, изолюпанина и их суммарное содержание. Выявленная изменчивость суммарного содержания алкалоидов в семенах изучаемой выборки составляла 0.0015–2.017 %. В большинстве случаев значение признака соответствует статусу образца: сорта современной селекции, за исключением сидеральных, входят в группу с показателями 0.0015–0.052 %, в то время как старые, местные сорта и дикие формы имеют значения 0.057–2.17 %. Характерно, что ко второй группе относятся преимущественно образцы, поступавшие в коллекцию до 1950-х гг., т. е. до периода активной селекции низкоалкалоидных сортов. Отмечена сильная межгодовая изменчивость признака у образцов, выращиваемых в одних и тех же почвенно-климатических условиях в течение двух лет. В 2019 г. в среднем по выборке содержание алкалоидов было в 1.9 раза выше, чем в 2020 г. Анализ погодных условий вегетации позволяет предположить, что снижение содержания алкалоидов произошло за счет значительного увеличения суммы осадков в 2020 г. При поиске связей содержания алкалоидов с типом боба (не вскрывающий- ся без обмолота – культурный, спонтанно вскрывающийся – дикий и промежуточный) наблюдается тенденция к более высокому (примерно в 2 раза в оба года исследования) суммарному содержанию алкалоидов у образцов с диким типом боба и приближенным к нему промежуточным по сравнению с не вскрывающимся без обмолота бобом. Связь среднего суммарного содержания алкалоидов с окраской семени, сведенной к трем категориям (темная – дикая, светлая – культурная и промежуточная), была достоверно выше у группы с темными семенами: в 5.2 раза в 2019 г. и в 3.7 раза в 2020 г. Не обнаружено достоверных различий процентного содержания отдель- ных алкалоидов в общей сумме алкалоидов как между годами исследования, так и между группами с различным типом боба и с разной окраской семени.

## Introduction

Narrow-leafed lupine (Lupinus angustifolius L., Fabaceae) is a species that has been cultivated as a crop for feed and food for less than 100 years. It was exploited for centuries as a green manure crop. Feeding the seeds of this high-protein plant to animals was possible only after soaking them in water with repeated water changes to extract antinutritional compounds – a complex of quinolizidine alkaloids. It was this feature that limited the use of the plant in fodder production, since alkaloids added bitterness to the feed and in high concentrations were toxic to animals and humans

The development of fodder cultivars was triggered by the discovery of low-alkaloid mutants (Sengbusch, 1931, 1942) and identification of the recessive mutations determining this trait: iucundus, esculentus, and depressus (Hackbarth, Troll, 1956). This event genetically underpinned the development of low-alkaloid forms and was regarded as the beginning of the species’ domestication (Gladstones, 1970). Nowadays, many fodder cultivars have been released for animal feed purposes and the possibility to use narrow-leafed lupine seeds in food production emerged (Vishnyakova et al., 2020).

The polymorphism of alkaloid content observed before the discovery of said mutants among wild forms of narrowleafed lupine was 0.4–3.0 % dry weight (DW) for seeds and 0.3–0.5 % DW for herbage (Święcicki W., Święcicki W.K., 1995; Brummund, Święcicki, 2011). After the release of numerous cultivars based predominantly on one iuc mutation, this polymorphism significantly increased. In a recent study by Polish scientists, who analyzed 329 lupine accessions, the character’s variability was recorded within the range from 0.0005 to 2.8752 % (Kamel et al., 2016). Currently, the threshold value for the content of alkaloids in seeds of food or feed lupine cultivars in a number of European countries and Australia is no more than 0.02 % DW (Frick, 2017). In Russia, the permissible level of alkaloid content ranges from 0.1 to 0.3 % DW for seeds of fodder lupine (State Standard R 54632-2011, 2013) and 0.04 % for food lupine seeds (according to the existing technical specifications developed by the Research Institute of Lupine (Specification No. 9716- 004-0068502-2008).

In routine practice, the content of alkaloids in seeds at the level of 0.05 % is considered the boundary value to distinguish between high-alkaloid (bitter) and low-alkaloid (sweet) lupines (Lee et al., 2007).

The content of alkaloids is very responsive to the impact of environmental factors, such as droughts, air temperature, geographic location, insolation level, agricultural practices, and the presence of pathogens (Christiansen et al., 1997; Cowling, Tarr, 2004; Ageeva et al., 2020). Moreover, the concentration of alkaloids in seeds of the same genotype under different growing conditions can show at least twofold variation, reaching even a tenfold increase, thus exceeding the required alkaloid content threshold and turning lupine genotypes traditionally classified as sweet into bitter ones (Cowling, Tarr, 2004; Reinhard et al., 2006; Romanchuk, Anokhina, 2018).

Along with a radical reduction of seed alkaloid content, the crop’s breeding improvement includes elimination of spontaneous pod dehiscence (opening) determined by the le (lentus) and ta (tardus) alleles, introgression of the genes responsible for early flowering and the absence of the need for vernalization (Jul and Ku ) into the genotypes of cultivars as well as the genes controlling seed coat permeability (moll – mollis), and white color of flowers and seeds (leuc – leucospermus) (Taylor et al., 2020).

The narrow-leafed lupine collection held by VIR includes 887 accessions from 26 countries. There are 261 cultivars developed by scientific breeding, 370 genotypes representing breeding material, 142 landraces and local varieties, 55 wild forms, and 50 accessions with an unclear status (Vishnyakova et al., 2021). The diversity of breeding statuses and the presence of wild relatives provide a rather motley picture of the presence/absence of domestication traits in the collection’s accessions. Many accessions have pods spontaneously dehiscent to various degrees, and all seed colors known for this species are present. Such versatility makes it possible to trace whether there are links among domestication traits in the accessions. Therefore, the objective of this study was to identify the degree of variability in the concentration of alkaloids in narrow-leafed lupine seeds under the impact of growing conditions during two years of research and analyze correlations of this character with seed color and the degree of spontaneous pod dehiscence in a set of accessions from the VIR collection.

## Materials and methods

Material. A set of 59 narrow-leafed lupine accessions from the VIR collection (Supplementary Material)1, grown in the experimental fields of VIR (Pushkin, St. Petersburg) for two field seasons (2019–2020), served as the material for this study. The set consisted of accessions from 20 countries included in the collection in different years and having different breeding statuses: scientifically improved cultivars, local varieties, breeding lines, and wild forms

Supplementary Materials are available in the online version of the paper: http://vavilov.elpub.ru/jour/manager/files/Suppl_Vishnyakova_Engl_27_2.pdf


**Fig. 1.**
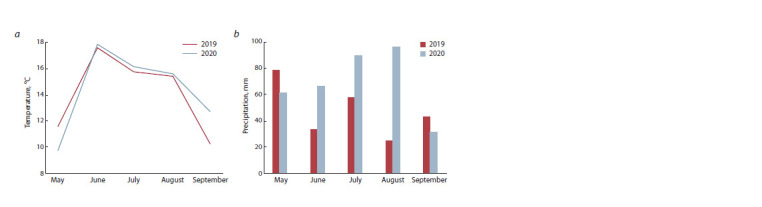
Weather conditions during the experiment: a – mean monthly air temperature; b – monthly precipitation amount

Weather conditions during the experiment. The sums of active temperatures amounted to 1966 °С in 2019, and 2052 °С in 2020. Precipitation amounts for the period with temperatures above 10 °С were 175 mm in 2019, and 293 mm in 2020. Mean values for the last 30 years (1992–2021) were 2209 °C and 306 mm, respectively. Thus, the years of research were cooler and drier than the long-term average. The precipitation amount during the active growing season in 2019 was lower by 118 mm, or 1.7 times, than in 2020, with a comparable heat supply. Differences between the years in the precipitation amounts were particularly significant during the pod ripening period: 58 mm vs. 91 mm in July, and 25 mm vs. 97 mm in August, respectively. Air temperatures and precipitation amounts by months are shown in Fig. 1.

Alkaloid content measurement in seeds. Each accession selected for the study was represented by 8 plants. An average sample (30 g) was taken from the mixture of seeds. The seeds were ground to flour (50–100 μm) in a Lab Mill 1 QC-114 (Hungary). The qualitative and quantitative compositions of alkaloids in narrow-leafed lupine seeds were assessed according to a previously published protocol (Kushnareva et al., 2020).

Ethyl acetate (8 mL) and 15 % NaOH solution (2 mL) were added to a 500 mg sample of flour and incubated at +6 °C for 18 hours. The resulting extract, containing alkaloids in the form of bases, was separated from the precipitate by filtration through a paper filter. A solution of caffeine in ethyl acetate (1 mg/mL) was used as an internal standard. The composition of alkaloids was analyzed using gas-liquid chromatography coupled with mass spectrometry on an Agilent6850 A chromatograph (Agilent Technologies, Santa-Clara, CA, USA). The mixture was separated on an AgilentHP-5MS capillary column (5 % phenyl, 95 % methylpolysiloxane; 25 μm). Heating program: +170 °C to +320 °C, heating rate: 4 °C/min. Temperature of the mass spectrometer detector: +250 °C, injector temperature: +300 °C, injected sample volume: 1.2 μL, carrier gas (helium) flow rate: 1.5 mL/min. Chromatogram recording started after 4 min, which was necessary for the solvent to exit, and continued for 38 min. The analysis was performed in three analytical replicates

Compounds were identified using the AMDIS program (Automated Mass Spectral Deconvolution and Identification System, National Institute of Standards and Technology, USA, Version 2.69, http://www.amdis.net). The NIST 2010 library (National Institute of Standards and Technology, USA, http:// www.nist.gov) was employed for the analysis

Alkaloid content was calculated according to the internal standard (caffeine, concentration: 1 μg/μL) using the UniChrom 5.0.19 program. The results of alkaloid content (absolute values) in narrow-leafed lupine seeds are given in mg/100 g DW. The percentage (%) of alkaloids (relative values) was calculated taking into account the proportion of an individual compound in the total alkaloid content, the latter being the sum of alkaloid values in an accession (mg/100 g DW). Mean values were calculated taking into account the resulting data of analytical replicates for each accession (see Supplementary Material).

The presence/absence of spontaneous pod dehiscence was assessed. It is better to describe this character shortly after harvesting, before the pods have reached the air-dry state, which provokes dehiscence even in such pods that were closed at the time of harvesting. Under our conditions, however, dry pods were assessed. On the one hand, it helped to reliably identify the type of pods nondehiscent without threshing; on the other hand, it hampered unambiguous identification of the pod opening time: whether the dehiscence of pods happened at harvesting or after complete drying. Therefore, this character was ranked according to the nature of the valves. The wild type (spontaneously dehiscent pods) had twisted valves (type 1). The cultivated type (nondehiscent pods) had flat valves, completely closed or slightly open (type 3). The remaining pods were open-valve, but flat or with some tendency to curl: they were classified into the intermediate type (type 2). A certain conventionality of the latter type and its closeness to the spontaneously dehiscent pod type should be recognized.

The seed coat color was also divided into three categories: dark (1), intermediate (2), and light (3).

Statistical processing. MS Excel programs and the Statistica 13.3 package (TIBCO Software Inc., USA) were used for data visualization. Statistical analysis was made in the Statistica 13.3 package

Statistical significance of differences in alkaloid content in 2019 and 2020 was studied using Student’s t-test for dependent (paired) samples (Dospekhov, 1973; Khalafyan, 2010). The difference in the characteristics of an accession in two versions of the experiment was calculated (in our case, between the years of research) and the significance of the mean difference of the accessions from zero was assessed using the t-test. Such criterion is more precise than a comparison of the differences between the means of independent samples, as it does not depend on the nature of the indicator’s distribution within the sample.

The average alkaloid contents in three groups of accessions with different pod types were compared using the analysis of variance; the same approach was applied for the groups with different seed colors. Correlation coefficients were calculated for alkaloid content separately in 2019 and 2020. The strength of correlations was assessed according to B.A. Dospekhov (1973): if the correlation coefficient is higher than 0.7 in its absolute value, it is strong; from 0.3 to 0.7, it is medium; less than 0.3, it is weak. The significance level of 5 % was adopted for this study.

## Results

Previously, the authors tested extraction techniques on alkaloids from leaves of the green manure cultivar Oligarkh (k- 3814) reproduced in 2018 (Pushkin) and clarified the qualitative composition of its alkaloid complex. The cultivar’s leaves contained five alkaloids: lupanine (L), 13-hydroxylupanine (H), angustifoline (A), sparteine (S), and isolupanine (I), plus traces of their derivatives or unidentifiable alkaloids numbering up to 120 in narrow-leafed lupine (Frick et al., 2017). The qualitative composition of main (detectable) alkaloids in seeds identified in the present study corresponded to our previous findings for vegetative organs. Their content in seeds varied as follows: 70.0–85.4 % for L, 6.4–17.2 % for H, 0.7–2.0 % for A, 4.0–12.6 % for S, and 0.5–1.4 % for I. The variability of the total alkaloid content was 0.0015–2.017 % (Table 1, see Supplementary Material).

**Table 1.**
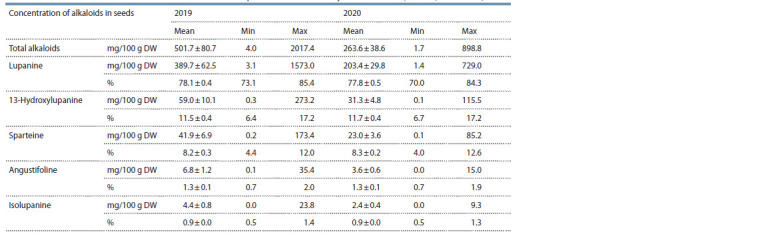
Mean alkaloid content in the set of 59 narrow-leafed lupine accessions for two years of research (Pushkin, 2019–2020)

The mean alkaloid level in the accessions was 501.7 mg/100 g DW in 2019, which was significantly (by 90.5 %) higher than the same value for the seeds reproduced in 2020 – 263.6 mg/100 g DW (statistical significance of differences according to Student’s t-test for dependent samples was p = 0.009). In 2020, a decrease in the mean alkaloid content values was observed: L was 389.7 in 2019 vs. 203.4 in 2020 ( p = 0.008); H: 59.0 vs. 31.3 ( p = 0.014); S: 41.9 vs. 23.0 ( p = 0.017); A: 6.8 vs. 3.6 ( p = 0.014); I: 4.4 vs. 2.4 ( p = 0.023) (see Table 1 and Supplementary Material).

However, the amount of alkaloids in eight accessions (k-3172, 3457, 3947, 3607, 3526, 1546, 2856, and 3062) increased in 2020 compared to 2019.

**Fig. 2.**
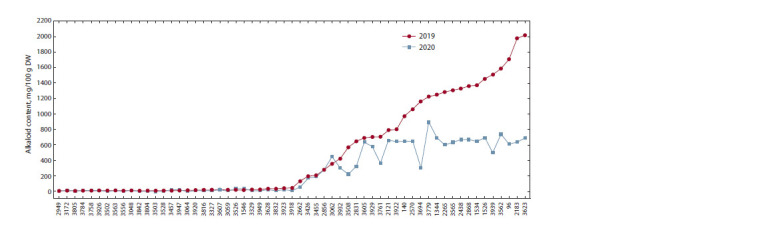
Alkaloid content in seeds of 59 narrow-leafed lupine accessions in the two years of research.

L content increased in 6 accessions, H in 13, S in 12, A in 9, and I in 10. It should be mentioned that, according to the International COMECON list of descriptors for the genus Lupinus L. (Stepanova et al., 1985), five accessions from this group were classified as having “very low” alkaloid content (its amount in seeds was less than 25 mg/100 g), two as “medium” (from 100 to 300 mg/100 g), and only one accession (cv. Oligarch, k-3814) had “very high” content (more than 300 mg/100 g). Characteristically, the accessions with very low or medium alkaloid content manifested insignificant differences across the two years: for example, k-2856 had 279.1 and 280.9 mg/100 g DW (0.7 %); k-3607, 21.2 and 23.2 mg/100 g DW (1.1 %); k-3172, 5.3 and 6.8 mg/100 g DW (1.3 %), respectively (Fig. 2).

The character’s low variability in low-alkaloid lupine forms, as observed above, was characteristic of all low-alkaloid accessions in the tested set, regardless of the increase or decrease in alkaloid concentrations across the years of research (see Fig. 2). Meanwhile, alkaloid content values remained within the established range: none of the sweet accessions with alkaloid content below 50 mg/100 g exceeded those values and did not shift into the bitter category. For example, the amount of alkaloids in cv. Yan (k-3832) was 7 mg/100 g in 2020 and 4.7 times higher (34 mg/100 g) in 2019.

Cv. Gerkules (k-3923) had 20 mg/100 g in 2020, and a more than twice higher amount in 2019 (47 mg/100 g), but in both cases the cultivar remained in the sweet category

On average for the studied set of accessions, the proportion of individual alkaloids varied insignificantly over the years. The relative content of L in 2019 and 2020 was 78.1 and 77.8 %; H: 11.5 and 11.7 %, and S: 8.2 and 8.3 %, respectively. The shares of A (1.3 %) and I (0.9 %) in the composition of alkaloids did not change during the period of research. Thus, the relative content of individual alkaloids may be recognized as a fairly constant indicator

A strong correlation was observed between the absolute content values (mg/100 g) of individual alkaloids: 0.89–0.96 in 2019 and 0.88–0.95 in 2020. The correlation between the amounts of individual compounds and total alkaloids was 0.94–0.999. The strongest relationship was observed between the total alkaloid content and L values: 0.999 in 2019 and 0.998 in 2020

Pairwise correlations between the percentage (relative) contents of the studied alkaloids were mostly insignificant; no systematic shifts in the alkaloid composition structure were observed over the years. A t-test for dependent samples showed a significance level of differences in the percentage of individual alkaloids: from p = 0.063 to p = 0.082. Variations of alkaloid composition in individual accessions were induced by changes in the representation of two main alkaloids, L and G: an increase in the proportion of one led to a decrease in the proportion of the other. Meanwhile, lupanine remained dominant in the composition of alkaloids in narrow-leafed lupine.

**Fig. 3.**
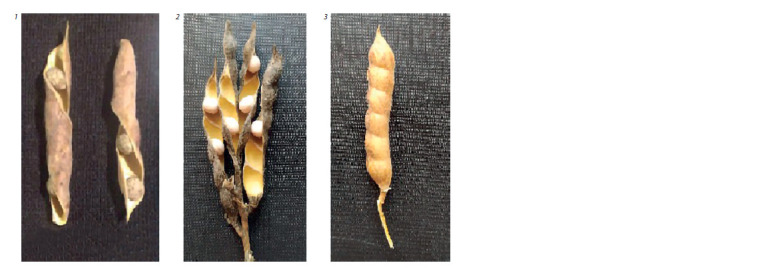
The types of narrow-leafed lupine pods according to their ability to dehisce spontaneously. Pod type designations: 1 – wild; 2 – intermediate; 3 – cultivated (nondehiscent without threshing).

**Fig. 4.**
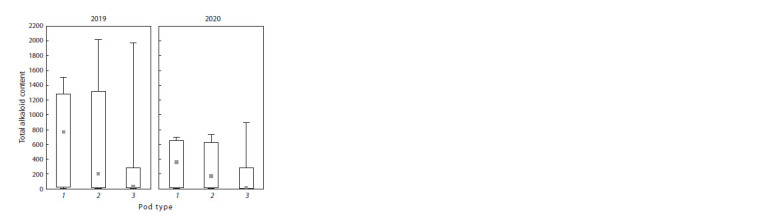
Total alkaloid content in lupine groups with different pod types in 2019 and 2020 Pod type designations: 1 – wild; 2 – intermediate; 3 – cultivated.

The types of pod dehiscence and seed coat color were analyzed for 45 accessions from the studied set. Twelve accessions were classified according to their pod characteristics into type 1 (wild, with twisted valves), 16 into type 2 (intermediate), and 17 into type 3 (nondehiscent without threshing) (Fig. 3 and Supplementary Material). There were no significant differences among the groups of accessions with different pod types in either the absolute or relative alkaloid con- tent (Table 2, Fig. 4). With this in view, in both years of research, the highest alkaloid content was recorded for type 1 (693.7 mg/100 g DW in 2019, and 345.3 mg/100 g DW in 2020), and the lowest, for type 3 (320.3 mg/100 g DW in 2019, and 200.1 mg/100 DW in 2020). Medium values were shown for type 2 (612.1 mg/100 g DW in 2019 and 300.7 mg/100 g DW in 2020).

**Table 2.**
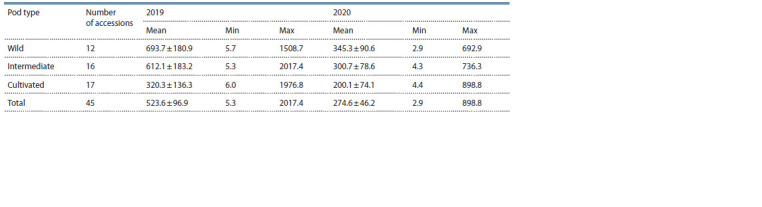
Total content of alkaloids in the groups of lupine accessions with different pod types

Consequently, higher total alkaloids were characteristic of the accessions with the wild pod type. They exceeded the accessions with the nondehiscent pod type 2.3 (2019) and 1.8 times (2020). However, taking into account the high variability in the absolute and relative content of individual alkaloids and their total amount, no significant differences were observed among the groups (see Table 2, Fig. 4). The contributions of individual alkaloids to the total content did not depend on the pod type.

According to the color of the seed coat, 15 accessions were characterized as dark-seeded, 19 were of the intermediate type (between dark and light), and 11 were light-seeded.

**Fig. 5.**
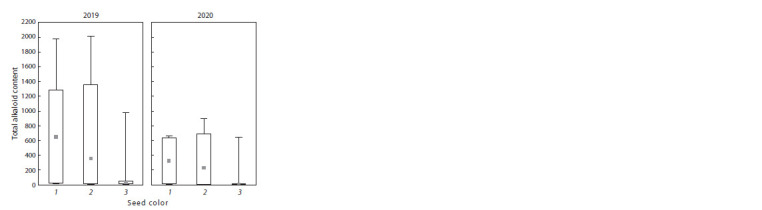
Total alkaloid content in lupine groups with different seed colors in 2019 and 2020. Seed color group designations: 1 – dark-seeded; 2 – intermediate; 3 – lightseeded

Differences in the studied traits among the above-mentioned groups were assessed as statistically significant at a 10 % significance level. In 2019, all three groups significantly differed from each other in the following parameters: L ( p = 0.063), H ( p = 0.066), S ( p = 0.070), I ( p = 0.075), and total alkaloids ( p = 0.062) (Table 3, Fig. 5); in 2020, in L ( p = 0.083), H ( p = 0.055), S ( p = 0.060), and total alkaloids ( р = 0.074). For A and I, significance levels of differences were 0.108 and 0.130, respectively. The highest values of the total alkaloid content were recorded for the dark-seeded group (660.4 mg/100 g DW in 2019 and 334.4 mg/100 g DW in 2020), while the lowest values were found in the light-seeded group (125.9 mg/100 g DW in 2019 and 90.6 mg/100 g DW in 2020) (see Table 3, Fig. 5).

**Table 3.**
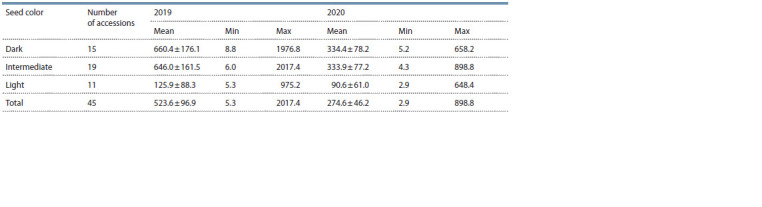
Total alkaloid content in the groups of lupine accessions with different seed colors

Significant differences at a 5 % level were observed between the 1st and 3rd contrasting groups of accessions: in 2019 for all indicators (L, H, S, A, I, and total alkaloids), and in 2020 for L, H, S, and total alkaloids. The dark-seeded group exceeded the light-seeded one in the mean value of total alkaloids 5.2 times in 2019 ( p = 0.023) and 3.7 times in 2020 ( p = 0.030).

Differences among the three color groups in the percent contribution of individual compounds to the total content of alkaloids were statistically insignificant ( p > 0.462). No significant differences were recorded between the groups contrasting in seed color (dark and light) during the period of research ( р > 0.237). Thus, lupine accessions with different seed coat colors significantly differ from each other in the total content of alkaloids, while their alkaloid composition can be recognized as constant

## Discussion

The analysis of alkaloid content in narrow-leafed lupine seeds from the VIR collection disclosed high variability of this character. In the selected set of 59 accessions, there were genotypes with minimum (0.0015 %) and maximum (2.017 %) values of this indicator: Cv. Danko from Belarus (k-2949) and an Australian breeding line (k-3623), respectively. The accessions were divided into two groups according to their alkaloid content: low-alkaloid (alkaloid content in seeds was below 0.05 %), and high-alkaloid (alkaloid content was equal to or above 0.051 %). The first group included 28 accessions, representing breeding lines and cultivars that entered the collection after 1950, mainly from Russia, Belarus, and Australia. The second group consisted of accessions received before 1950: local varieties, improved cultivars and lines from Germany, United Kingdom, Poland, and Latvia. In addition, it included wild genotypes from Greece and Spain.

It should be mentioned that in the high-alkaloid group there were several improved cultivars of contemporary breeding, for example, cv. Oligarkh (k-3814, Leningrad Research Agriculture Institute) grown for green manure, and the Australian breeding line (k-3623) also, apparently, developed for green manure purposes. Cultivars intended to be used as green manure have high vegetative weight and, as a rule, low seed productivity. These features were observed in cv. Oligarkh, characterized by rapid initial growth, early maturation, and good leafiness, which ensures high yields of green biomass and readiness for plowing 50–60 days after sowing (Lysenko, 2020). Such cultivars are usually developed without any regard to the content of alkaloids, and they may appear nonuniform in this indicator. Sporadic low-alkaloid genotypes, in their turn, occur among wild lupine forms, for example, in accessions k-3607 (Spain) and k-3457 (Greece), the alkaloid content of which did not exceed 0.021 %, thus classifying them as sweet forms of narrow-leafed lupine.

Our data are quite in agreement with the results obtained by Polish scientists who screened the collection of narrow-leafed lupine maintained at the Polish genebank and found low- alkaloid genotypes in the group of wild accessions where the range of this character was 0.0163–2.8752 % DW. Contrariwise, high-alkaloid accessions were identified among cultivars developed by scientific breeding, for example, cv. Karo (1.165–1.3011 %). The character’s range in this group was 0.0022–2.1562 % DW (Kamel et al., 2016).

The variation of this character between the years of our experiment manifested itself in the fact that in 2020 the average alkaloid content in the studied set was 1.9 times lower than in 2019

The high susceptibility of alkaloid content levels in lupine seeds to environmental factors has not yet been explained. The mechanisms of their impact are even called unpredictable (Frick et al., 2017). As mentioned previously, alkaloid content variability in the same genotype can be affected by a variety of environmental factors: temperature, humidity, soil characteristics and mineral composition, geographic location, etc. The amplitude of the character’s variability also depends on the genotype: some cultivars are more variable under environmental impacts than others (Gremigni et al., 2001; Cowling, Tarr, 2004; Jansen et al., 2009).

Plants in this experiment were grown for two years in the same location, on a field relatively homogeneous in soil composition, and the same agricultural practices were used. Therefore, we consider weather conditions to be the main factor that could affect the content of alkaloids. The most significant meteorological differences across the two years of research manifested themselves in an increase in the precipitation amount in 2020 compared to 2019 (see Fig. 1). A particularly noticeable rainfall deficit was observed in July and August of 2019 (58 mm and 25 mm, respectively). These are the months when seeds are swelling and ripening and alkaloids from vegetative organs accumulate in them (Vishnyakova, Krylova, 2022). Apparently, it was this factor that led to a decrease in total alkaloids on average for the studied set of 59 accessions from 501.7 mg/100 g DW in 2019 to 263.6 in 2020.

Droughts are believed to increase the content of alkaloids in lupine, but it is important at what stage of plant development the drought occurs (Christiansen et al., 1997). An increase in the level of alkaloids under drought conditions was observed in a number of plant species: Nicotiana, Papaver somniferum, and Catharanthus roseus (Waller, Nowacki, 1978; Szabó et al., 2003; Jaleel et al., 2007; Amirjani, 2013). Stresses are presumed to increase the synthesis of secondary metabolites, such as isoprenoids, phenols, and alkaloids (Selmar, Kleinwächter, 2013). In view of this, temporary exposure to drought is recommended to intensify the synthesis and increase the yield of alkaloids in medicinal and spicy herbs (Kleinwächter et al., 2015; Kleinwächter, Selmar, 2015).

High air temperatures from the start of flowering to pod maturation are also considered to be a factor raising alkaloid concentration in narrow-leafed lupine (Jansen et al., 2009). Under the conditions of our experiment, the year 2019, when the accumulation of alkaloids was at its peak, was on the whole much colder than either 2020 or the long-term mean value, but the temperatures during the growing season in both years were comparable. Therefore, we consider precipitation to be the decisive factor in the variation of this character across the two years of research.

No significant differences in the percentage of individual alkaloids within their total amount were found between the years of research. Their average contribution was as follows: 77.9 % of lupanine, 11.6 % of 13-hydroxylupanine, 8.3 % of sparteine, 1.3 % of angustifoline, and 0.9 % of isolupanine

The almost twofold increase in alkaloid concentration, averaged for the studied set of accessions, was typical only for high-alkaloid and medium-alkaloid genotypes. For accessions with alkaloid content less than 0.05 %, this indicator changed relatively little in both years. It is quite possible that these accessions reached the lowest alkaloid accumulation threshold for narrow-leafed lupine seeds. In any case, these very low-alkaloid genotypes can be regarded as stable in the manifestation of the trait

Spontaneous pod dehiscence is one of the key features differentiating wild species from cultivated ones in legumes. During pod maturation and drying, the valves of wild genotypes suddenly spontaneously open along the dorsal and ventral sutures and rapidly twist along their axis spirally in opposite directions, giving the opened pods a typical V-shaped appearance (Maysuryan, Atabekova, 1974). Wild species use this mechanism to disperse seeds, while in cultivated plants it is a highly undesirable trait that leads to yield loss Contemporary breeders, along with the efforts to develop alkaloid-free narrow-leafed lupine cultivars, make attempts to introgress as many other domestication genes into their genomes as possible, specifically those responsible for the absence of spontaneous pod dehiscence. This trait is known to be controlled by two recessive alleles: ta (tardus), which determines the fusion of pod valves by forming a solid strand of sclerenchyma cells along the pod’s perimeter (Hackbarth, Troll, 1959), and le (lentus), which changes the orientation of endocarp cells and reduces the thickness of the parchment layer (Gladstones, 1970). Only the combination of both alleles can ensure complete absence of spontaneous pod dehiscence (Anokhina et al., 2012). It is quite possible that the accessions classified by us into the intermediate pod type according to their pod dehiscence nature possess only one of these two alleles. This study pinpointed a quite obvious tendency towards higher alkaloid content in the accessions with wild-type pods and intermediate ones that were close to the wild type, compared to cultivated genotypes with nondehiscent pods. They demonstrated an almost twofold difference in both years of research.

A similar relationship was observed between the content of alkaloids and the color of seeds (seed coat). There are up to 8 grades of seed coat color recognized in narrow-leafed lupine: (1) variegated, gray, with indistinct maculation; (2) almost black, with small white speckles and spots; (3) gray with white spots; (4) white with occasional brown and gray spots; (5) beige (nut-brown), with brown spots; (6) white, dull at the hilum, without a triangular spot or a stripe; (7) white, with sporadic brown spots; and (8) pure white, glossy. (Kurlovich, 2002). Similarly to the pod dehiscence pattern, we reduced these grades to three types: (1) dark, or wild type included seeds of the 1st and 2nd seed color grades, (2) intermediate type, with the 3rd and 5th seed color grades, and (3) light, or cultivated type, incorporating the 4th, 6th, 7th and 8th color grades

It is known that wild forms of narrow-leafed lupine have blue flowers and dark seeds. Breeders, in their efforts to improve this crop, selected plants with the leucospermus locus, responsible for the white color of flowers and light-colored seeds (Nelson et al., 2006; Berger et al., 2012). The same pattern was also observed in the domestication of other legume species (Ku et al., 2020). In our study, the group with dark seeds significantly exceeded the group with light seeds in the average total content of alkaloids (5.2 times in 2019, and 3.7 times in 2020). Meanwhile, no such differences were found in the percentage content of individual alkaloids either among the groups of accessions with different seed coat colors or those with different pod types.

Thus, low alkaloid content in narrow-leafed lupine seeds, acquired by a part of the crop’s gene pool as a result of domestication and breeding improvement, is associated with the absence of spontaneous pod dehiscence and the light color of seeds. We regard this phenomenon as the evidence of the joint introgression of domestication genes into modern narrowleafed lupine cultivars.

## Conclusion

Development of low-alkaloid narrow-leafed lupine forms, i. e., reducing the concentration of alkaloids in lupine seeds to a level below 0.05 %, has been a priority trend in the species’ improvement in the process of domestication and breeding.

The VIR collection contains cultivars for feed and food uses with the content of alkaloids no higher than 0.0015 % DW. It is this minimum value that we found while screening the set of 59 accessions. Susceptibility of this trait to the impact of environmental conditions was seen in the fact that the synthesis of alkaloids in 2019 was 1.9 times more intensive than in 2020 on average for the studied set of accessions. A significant precipitation deficit was recorded in July and August of 2019, with all other growing conditions being comparable. This stressor, apparently, was the decisive factor that provoked an abrupt increase in the synthesis of alkaloids compared to 2020.

A distinctive feature of alkaloid content variability in narrow- leafed lupine seeds under the impact of environmental conditions is relatively low variation of this character in lowalkaloid genotypes

The observed tendency towards higher (almost twofold) alkaloid content in the accessions with spontaneously dehiscent pods than in those with pods nondehiscent without threshing in both years of research and significantly higher content of alkaloids in seeds with dark seed coat color (wild) attest to the joint introgression of these domestication traits into modern cultivars

## Conflict of interest

The authors declare no conflict of interest.
